# Supplementary material for: A Likelihood Approach for Real-Time Calibration of Stochastic Compartmental Epidemic Models
Source: PLoS Comput Biol. 2017 Jan 17;13(1):e1005257. doi: 10.1371/journal.pcbi.1005257 (PMC5240920; doi:10.1371/journal.pcbi.1005257)
Supplement: S1 File — (TAR.GZ) [file pcbi.1005257.s014.tar.gz › HSPH_Online-SI-Revision/Additions/MSS11/n100-extreme-SEITR/n100-extreme-SEITR_NEW2_summary2.pdf]

peak -8	3w spec	3w cum	5w spec	5w cum	end	3w spec LOG	3w cum LOG	5w spec LOG	5w cum LOG	end LOG
mean	258.7	222.7	81.3	110.9	25.6	0.7	0.6	0.9	0.8	0.4
median	70.1	64.5	76.5	73.1	17.9	0.4	0.3	0.5	0.5	0.1
quantile .1	19.3	16.7	20.6	18.6	8.7	0.1	0.1	0.1	0.1	0.
quantile .9	347.4	321.6	119.1	144.4	44.6	1.8	1.3	2.6	0.1	1.3
23	24	22	22	3						

peak -4	3w spec	3w cum	5w spec	5w cum	end	3w spec LOG	3w cum LOG	5w spec LOG	5w cum LOG	end LOG
mean	15.4	12.8	27.4	16.9	24.	0.1	0.1	0.2	0.1	0.1
median	12.9	11.4	22.1	14.7	22.4	0.1	0.1	0.1	0.1	0.1
quantile .1	3.6	3.7	8.4	4.3	6.8	0.	0.	0.	0.	0.
quantile .9	26.3	24.9	52.6	29.1	43.5	0.1	0.1	0.3	0.	0.2
15	22	2	5	0						

peak	3w spec	3w cum	5w spec	5w cum	end	3w spec LOG	3w cum LOG	5w spec LOG	5w cum LOG	end LOG
mean	26.5	19.3	36.5	21.8	24.2	0.1	0.1	0.2	0.1	0.1
median	27.	18.6	39.9	21.9	25.1	0.1	0.1	0.2	0.1	0.1
quantile .1	12.5	11.7	8.5	13.3	9.4	0.1	0.1	0.	0.1	0.
quantile .9	39.	29.2	53.1	30.5	37.1	0.2	0.2	0.3	0.1	0.2
0	0	1	0	0						

peak +4	3w spec	3w cum	5w spec	5w cum	end	3w spec LOG	3w cum LOG	5w spec LOG	5w cum LOG	end LOG
mean	56.8	52.3	67.1	53.3	54.2	0.6	0.5	0.6	0.5	0.5
median	55.4	49.6	63.6	54.1	55.5	0.3	0.3	0.4	0.3	0.3
quantile .1	19.8	20.4	14.4	20.8	17.1	0.1	0.1	0.1	0.1	0.1
quantile .9	96.8	90.2	99.9	90.6	90.9	1.1	1.	1.2	0.1	1.
1	0	3	0	1						

peak +8	3w spec	3w cum	5w spec	5w cum	end	3w spec LOG	3w cum LOG	5w spec LOG	5w cum LOG	end LOG
mean	224.9	100.	483.7	101.5	102.6	1.8	1.7	1.7	1.7	1.7
median	99.6	99.5	99.8	99.5	99.6	2.	1.9	1.7	1.9	1.9
quantile .1	32.5	37.3	33.3	34.6	32.3	0.2	0.2	0.1	0.2	0.2
quantile .9	100.	99.9	100.	99.9	99.9	3.	2.8	2.9	0.2	2.8
2	0	4	0	0	{}	{}	{}	{}	{}	{}
